# SDG3 sustainable development model for Chinese ethnic intangible cultural heritage sports projects: a quantitative study

**DOI:** 10.3389/fspor.2025.1651771

**Published:** 2025-10-28

**Authors:** Jiangping Fu, Hua Li, Yi Yu, Ninggang Yue, Weiping Zhang, Tian Zhou

**Affiliations:** ^1^ Nantong Institute of Technology, Nantong, China; ^2^ Sports School, Sichuan Technology and Business University, Sichuan, China; ^3^ Jiangsu Open University, Nanjing, China; ^4^ Sports School, Nantong Normal College, Nantong, China; ^5^ Sichuan University, Sichuan, China

**Keywords:** sustainable development, ethnic intangible cultural heritage, sports culture, China, risk factors, integrated mode, quantitative

## Abstract

**Objective:**

Background: The World Intellectual Property Organization (WIPO) 2022 report emphasizes that 75% of intangible cultural heritage projects worldwide face the “modernization trap”, with the intensity of risk positively correlated with the degree of commercialization of the project. Sports projects should avoid this concrete phenomenon. The sustainable development of China's ethnic intangible cultural heritage sports projects must be based on a comprehensive/integrated model that encompasses sports culture (culture, economy, society, and resources).

**Method:**

Using integrated model theory, a sustainable development model was created. A group of 10 experts was established, and data was obtained through two rounds of questionnaires administered to the expert group. Through sports culture theory, combined with the EGG model (aggregation model), identify risk factors for safety accidents, identify risk factors for the lack of sports culture (intangible cultural heritage), and identify the relationship between the two types of risks. Based on a multi-subject collaboration mechanism, the Decision-Making Trial and Evaluation Laboratory (DEMATEL) is incorporated as a multi-criteria decision-making method to calculate the prominence and correlation of influencing factors and assess the degree of impact of risk factors.

**Results:**

A fault tree analysis of 54 indicators (risk factors) was conducted to determine a model for the sustainable development of intangible cultural heritage sports projects (EGG), including 5 first-level indicators, 11 s-level indicators, and 38 third-level indicators. The most prominent and influential factors (risk factors) that have been identified include five third-level indicators and one first-level indicator. Risks level-3 indicators) are divided into 5 major prominent causes/factors (T5, T6, T17, T23, and T34), and 4 major related causes/factors (T3, T7, T20, T33, and T36). The F5 intangible cultural heritage (sports) dimension, as an important dimension of the sustainable development model (representing well-being), was observed (third prominent indicator, not last place; the lowest related causes/factors; sustainable well-being is not yet apparent).

**Conclusion:**

The results indicate that quantitative (calculation) integrated models are scientific and reliable tools, which can be used to reduce the risk assessment of sustainable development in Chinese ethnic intangible cultural heritage sports projects.

## 1 Introduction

China's strategy to become a sporting powerhouse has given rise to demands for the globalization of sports culture (1). In line with the fundamental spirit of the United Nations 2030 Agenda for Sustainable Development (2), the globalization of sports culture must be led by national intangible cultural heritage sports projects, bringing good health and well-being to people. The World Intellectual Property Organization (WIPO) 2022 report (Page 86) emphasizes that 75% of intangible cultural heritage projects worldwide face the “modernization trap”, with the intensity of risk positively correlated with the degree of commercialization of the project. The predicament of sports projects should avoid this concrete phenomenon (3). China's ethnic intangible cultural heritage sports projects (CEICHSP), which is intangible cultural heritage projects in the field of sports that embody traditional Chinese culture, have cultural symbol value in the construction of a sports powerhouse, but their modernization is facing systemic risks, especially excessive commercial development, which has led to a triple paradox (lack of cultural identity, low local community participation, and intellectual property confusion), Such as Tang Dynasty culture (4). The traditional risk management framework is no longer adequate to address the complex risk landscape of intangible cultural heritage sports. There is an urgent need to establish an adaptive governance system, including comprehensive models/integrated models (CM/IM). Therefore, this study proposes a CM/IM for assessing CEICHSP.

The World Bank's 2021 Cultural Heritage Risk Assessment Framework shows that the “risk chain reaction” model has a destructive force that grows 4.7 times within a 3–5-year window (5). CEICHSP risks exhibit cross-sector transmission characteristics, and a single risk may trigger a systemic crisis. Traditional risk management tools have significant limitations when dealing with the complexity of CEICHSP (6). CM/IM is a novel and feasible approach to promote sustainable development in response to the comprehensive risks of CEICHSP (7).

Cultural identity is the core driving force behind CEICHSP's continued existence (8). Living heritage emphasizes the ability of CEICHSP to continue in dynamic practice (9). These two are the core characteristics of CEICHSP's cultural sustainability (8, 9). In addition to cultural characteristics, CEICHSP's sustainability also encompasses three aspects: environment (10), society (11), and economy (12). Therefore, the sustainable development of CEICHSP is a complex multi-subject system.

Past and current research evidence indicates that cultural safety is a spatial concept (inheritance of intangible cultural heritage projects, emphasizing cross-cultural differences as risk factors/incidents), enhancing the resilience of intangible cultural heritage (cultural identity and living transmission), and promoting inclusivity and equity in public health (13, 14). The Egg Aggregated Model (EAM) is a safety science framework rooted in organizational behavior studies (15). It is employed to elucidate qualitative relationships among variables within organizational systems (16) and is claimed to be applicable across disciplines (17). Thus, EAM may serve as an effective approach for systematically and vividly describing the cultural security (risks/factors) of sustainable development in intangible cultural heritage sports projects. Based on system dynamics, FTA is an important and widely used method for identifying hazards (top-level events) and causal relationships (incidents and system components) (18, 19). The sustainable development of cultural safeguarding for intangible cultural heritage sports projects, based on the EAM and FTA approaches, requires ongoing attention.

In order to quantitatively assess the comprehensive risks to the sustainable development of CEICHSP, this paper focuses on a quantitative CM/IM, based on system dynamics theory/complexity theory and multi-subject coordination theory (20–22). Our research objectives mainly include (1): Design/build a sustainable CM/IM (structure and factors) for risk/safety assessment. (2) Steps for risk/safety assessment of sustainable development using integrated models. (3) Quantitative algorithms to identify the causes (risk factors) of sustainability risks/incidents in the EAM and FTA system. (4) Quantitative algorithms are used to identify complex risk interactions (Classified by Risk Category) for decision-making management mechanisms.

## 2 Methods

### 2.1 Research design

The research design of this paper uses quantitative methods to assess the relationship between CM/IM and risk factors for sustainable development model (SDM) in CEICHSP. Our study was approved by the Institutional Review Board (NIT-2024PE-008).

We formed research working group to control the reliability and validity of the overall research process. The research team consisted of all five authors of the study, four of whom hold PhD degrees and three of whom are associate professors. Three of them are CEICHSP research project leaders, and one is a CEICHSP disseminator. The research team also invited an expert in mathematics to help researchers control the accuracy of mathematical calculations in the study. The research team worked together in an equal manner, collaborating randomly according to the first author's work procedures to complete the research task.

**Figure 1:**
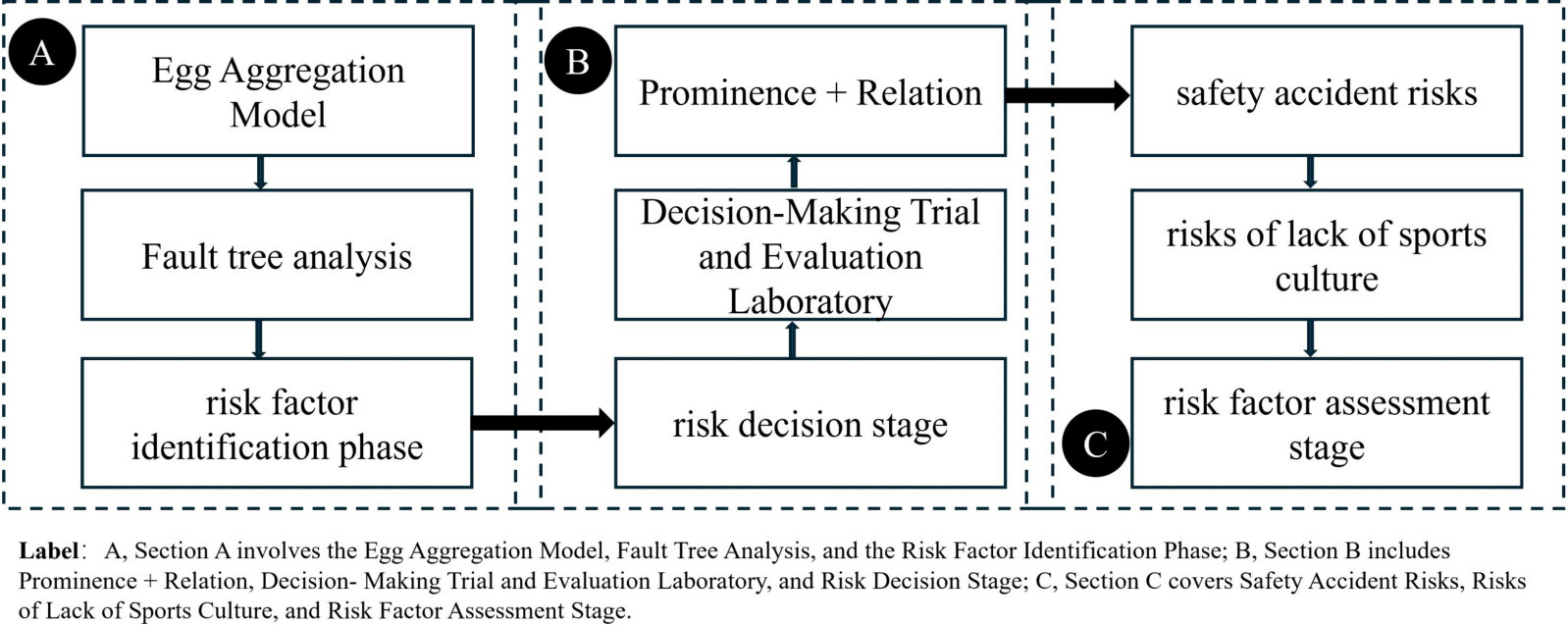
Sustainable development (Integrated/comprehensive) model.

In order to quantitatively assess the comprehensive risks to the sustainable development of ethnic intangible cultural heritage sports projects, this study proposes a CM/IM (risk/safety) model, as shown in Figure 1. Throughout the CM/IM, CEICHSP's risk assessment study was divided into three phases:

The first stage [Figure 1A] is the risk factor identification phase, with the primary task of incorporating the Egg Aggregation Model (EAM) to systematically and comprehensively describe the sustainable development system (23); Then, fault tree analysis (FTA) is incorporated (24), combined with sports culture, to determine the topological structure of safety incident (risk) factors (human, equipment, environment, and management dimensions) and intangible cultural heritage (sports) cultural (risk) factors (physical activity and competition performance), systematically and comprehensively explaining the relationships (causes) between risk factors in the sustainable development system. A systematic and comprehensive risk factor represents an expression of the entire causal system. A detailed and clear expression entails that the risk factor encompasses all levels of indicators (Level 1, Level 2, and Level 3).

The second stage [Figure 1B] is the risk decision stage, whose main task is to implement decision management mechanisms (quantitative algorithms), incorporated into the Decision-Making Trial and Evaluation Laboratory (DEMATEL) to quantify complex risk interactions (Prominence and Relation), and determine the causes of further modeling (CM/IM) (18).

The third stage [Figure 1C] is the risk factor assessment stage, whose main task is to analyze the risk factors for sustainable development, including a vertical analysis of safety incidents (risk factors) and a horizontal analysis of sports culture (risk factors), to assess the scientific nature and effectiveness of SDM.

### 2.2 Research materials (EAM, FTA and questionnaire)

EAM is the basis of the entire study and can systematically and comprehensively explain the concretization of SDM. It is a subsystem of CM/IM. Safety behaviors in sports culture based on human dynamics are the logical starting point behind CEICHSP's EAM system (23). EAM provides a complete and clear structure and relationship, divided into the easiest part to observe (egg yolk), the more difficult part to observe (egg protein), the most difficult part to observe (air), and the barrier part (eggshell).

FTA is a method for determining the topological relationships within the internal structure of an EAM system (24). Based on system dynamics, FTA is an important and widely used method for identifying hazards (top-level events) and causal relationships (incidents and system components) (19, 25). Based on the established internal structure of EAM, this paper uses hierarchical methods and binomial classification methods to determine the accident factors of SDM at three levels (26, 27).

The questionnaire is the implementation tool for this study. Following the work of the research team on EAM and FTA, a comprehensive survey questionnaire (CEICHSP SDM Assessment Questionnaire) was developed.

The first part of the questionnaire covers basic demographic information, including gender, age, role, education level, length of service, professional qualifications, and intangible cultural heritage projects. The roles include managers/heirs/promoters, trainers/athletes, teachers/coaches, and referees. Among them, professional qualifications include ungraded, junior, intermediate, and senior levels. Our study does not intend to statistically analyze demographic variables or add basic information, but only to ensure the validity and reliability of the study and the reliability of the sample selection.

The second part of the survey questionnaire is a standard Likert scale questionnaire. This version of the questionnaire consists of four sections and 38 items, which are derived from the accident factors identified by the FTA. This version of the questionnaire uses a five-point Likert scale for scoring (1 = extremely likely, 2 = likely, 3 = occasionally, 4 = unlikely, 5 = very unlikely; total score ranges from 18 to 225) (28).

A project portfolio was identified through meta-analysis and meta-synthesis methodologies.

The research group prepared a project pool by searching five major databases (China National Knowledge Infrastructure, ERIC, ScienceDirect, Scopus, and Sport), using the keywords intangible cultural heritage, sports management, and risk management (risk factors).

It is well known that safety/accidents fall within the scope of research in both industrial settings and sports. In the field of public health, new research (safety culture) has been directed toward the humanistic qualities of medical personnel and patients (29).

Building upon established models for laboratory safety incidents (30) and sports competition safety (31), the research group incorporated public health literacy (32) to establish a project pool. Our study aims to awaken shared values of safety culture among members (both administrators and participants) of cultural and sports organizations, through the sustainable development of intangible cultural heritage sports.

Based on meta-analyses and meta-syntheses, the research group ensured the validity and practicality of their study by establishing a framework document to analyze the concept of safety culture (risk factors) and conducting pilot tests.

The cross-cultural translation work between English and Simplified Chinese was completed by three researchers from the study team. The first researcher initially handled the translation from English to Simplified Chinese. The second researcher was responsible for the reverse translation from Simplified Chinese to English. The third researcher oversaw the bidirectional review of both English and Simplified Chinese translations.

Pilot testing was conducted by the research team, who invited 90 members from university-based intangible cultural heritage interest groups (30 per project) to participate in pilot testing of the project pool based on the distinct characteristics of the three intangible cultural heritage projects. The 42 entries in the project pool (each risk concept described in a single sentence) achieved 100% agreement (all 90 participants deemed the concepts clear). The pilot testing process was completed in three separate sessions, each led by a different researcher. During the pilot testing, researchers distributed framework documents on-site, read and introduced each item individually, and then had participants complete the assessment (selecting 1 for clear and 2 for unclear). This pilot testing ensured the risk factors (concepts) in the research were scientifically sound and rigorous (if university student participants could perceive the clarity of the concepts, experts should also be able to understand their meaning).

The third part of the questionnaire consists of semi-open questions. Respondents can fill in (additional) incident factors as a supplement to the relevant items in Part 2 if they believe that there are no items in Part 2, and then score them.

### 2.3 Research sample and data collection

The research sample for this paper comes from regional populations participating in intangible cultural heritage sports projects (such as flower basket tossing, Hai'an flower drum, and Tang Dynasty sports), and role restrictions were applied in the survey questionnaire. We refer to the group of people who meet the role requirements as the expert group interviewees. The data collected from the expert group is mainly used as prior probability (data) and for quantitative modeling calculations.

Based on empirical rules and considering the 10 events per candidate predictor parameter (EPP) requirements, combined with the study's specific characteristics (33 items, all-cause relationships of risk factors), a C statistic of 0.8–0.9 was determined. with an event proportion of 0.1–0.5 (33). This corresponds to 30 parameters (the manuscript has 33 parameters), requiring 8–15 10EPP. Since the event probability for the manuscript's respondents (experts) is 1, the actual value needed for our study is 8–15 (sample size, number of experts).

Three rounds of questionnaires were distributed and collected from the expert group and research team (the respondents were the same, completed the research procedures for the expert consensus method.).

The first round will begin in November 2024. Questionnaires (QR codes) were distributed to 10 high-quality expert groups. One researcher was assigned to collect the completed questionnaires within three days. In the second round, after the first round of samples are collected, if additional items are found, a research team meeting will be held to confirm whether they need to be added/deleted. If it is confirmed that they need to be added/deleted, the questionnaire will be modified and distributed in the second round. Otherwise, the results of the first round will be used. The second round will still include the third part of the questionnaire. In the third round, after the second round of sample collection, repeat the work of the second round, but the third-round questionnaire no longer includes the third part of the survey questionnaire; otherwise, continue the study based on the results of the second-round questionnaire.

The interval between each round of questionnaires was at least four weeks, ensuring the reliability and stability of the study. The research group developed inclusion criteria for additions/deletions in the second part of the questionnaire (risk factors, items), achieving 100% approval from all expert respondents—meaning each expert endorsed every single item. The proposed inclusion criteria were based on the study's objective: to examine the interrelationships among all risk factors, within the sustainable development model for intangible cultural heritage sports projects. This aims to achieve public health awareness and safety consciousness regarding accidents/risks among all stakeholders (managers/heritors/promoters, practitioners/athletes, teachers/coaches, and referees).

To ensure the reliability and validity of the samples and survey questionnaires, scientific preparatory work was conducted: (a) The research objectives were explained in the preface section of the survey questionnaire; (b) The survey questionnaire was anonymous, and consent was obtained from each respondent. Once the questionnaire was completed, it was deemed that informed consent had been obtained; (c) Respondents were informed that they could stop answering at any time. All data was used solely for scientific research purposes and was kept confidential, appearing only in statistical form and not containing any personal information; (d) The survey questionnaire was created, distributed, and collected via an online document (Wenjuan Xing); (e) All respondents were selected through a two-part process: the first part involved three intangible cultural heritage research project leaders recommending 10 experts, and the second part involved the recommended 10 experts further recommending 10 experts through a snowball sampling method. The research team then finalized the list of 10 experts as respondents.

Clear articulation of snowball sampling methodology: The research group created an expert referral form for use in the expert snowball referral process. This further ensured the rigor of the study. The expert referral form included all items from the first section of the questionnaire (basic information section), plus the names and contact details (phone number, email address, and WeChat ID) of both the referrer and the referred individual.

The precise criteria for including the final 10 expert respondents: After completing the collection of expert recommendation forms, the research group created selection criteria to determine the final list of high-quality respondents, who met the study's objectives and represented the full range of risk factors for the sustainable development model. The selection criteria for the high-quality expert group comprised a prioritized sequence of fundamental information, which is intangible cultural heritage project—role—professional qualifications—educational background—years of experience—age—gender.

### 2.4 Data calculation

**Figure 2:**
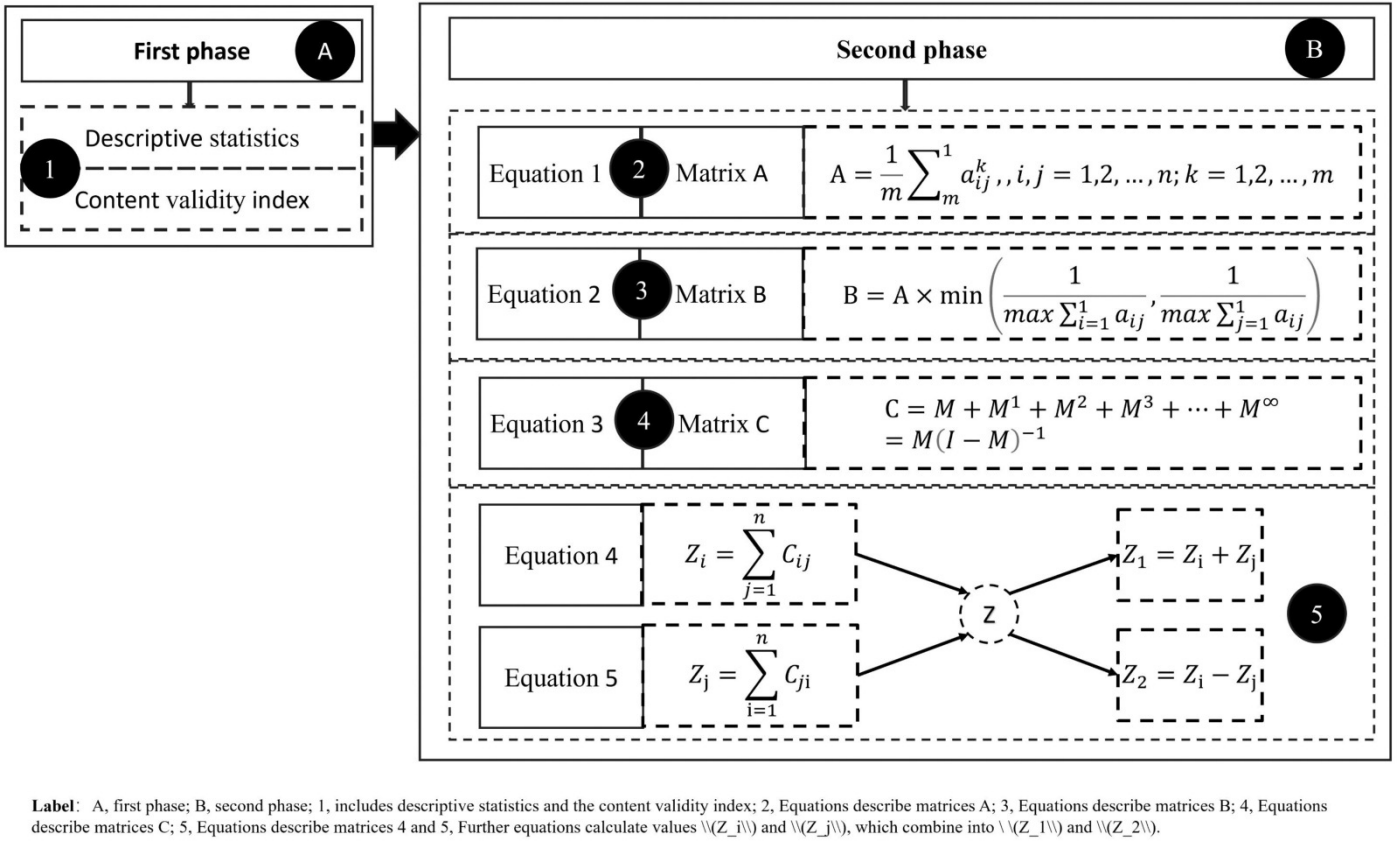
Calculation steps for sustainable development models.

Our data calculation mainly consists of two stages and five steps. See Figure 2.

(4) $$Z_{i}=\sum_{\;j=1}^{n}C_{ij}$$

(5) $$Z_{j}=\sum_{i=1}^{n}C_{\;ji}$$

#### 2.4.1 Descriptive statistics

The first stage involved descriptive statistics of basic demographic information from the survey questionnaire and calculation of internal consistency of the survey questionnaire items. See Figure 2A.

Descriptive statistics and content validity index (CVI) calculations are the first sub-steps of the overall calculation.

The computational tool for the first stage is Jamovi 2.6.26 (34).

The content validity index (CVI) of the survey questionnaire was assessed using Cronbach's alpha coefficient (>0.7, acceptable) to calculate the item-level (I-CVI ≥ 0.8, acceptable) and scale-level (S-CVI ≥ 0.9, acceptable) (35).

Kappa (K) index for content validity, was included to eliminate the possibility of chance agreement (≧0.40, acceptable) (36).

The Kendall W test incorporated into the expert consensus method validated the degree of agreement among all experts regarding risk factors (*p* < 0.05 indicates significant agreement; W ≥ 0.7 denotes high agreement, 0.3 ≤ W < 0.7 indicates moderate agreement, and W < 0.3 signifies weak agreement) (36).

#### 2.4.2 DEMATEL calculation

The second stage involves calculations related to risk decisions, including four sub-steps from 2 to 5 of the overall calculation.

Our study involved multiple subjects (role variables in the questionnaire: managers/heirs/disseminators, etc.), so this paper incorporated the DEMATEL method (multi-criteria decision-making) to calculate the interaction between risk factors (37). The prominence and correlation of each accident factor are transformed into causes and results of risk/safety to facilitate risk decision-making and complete the second stage of assessment.

The research team identified the logic behind the use of DEMATEL, which is divided into four sub-steps. See Figure 2B.

The second sub-step of the overall calculation is to determine the matrix A of incident factors. Matrix A is calculated by averaging the total scores for each item (each risk factor) in the questionnaire to obtain a matrix of average values. The matrix represents the direct relationship between risk factors, and the calculation logic is given by Equation 1. Equation 1 expresses that m is the number of experts, *n* is the number of risk factors (factors) in the questionnaire, and K represents the influence (score) of risk factor (factor) I on risk factor (factor) J as determined by the kth expert.

The third sub-step of the overall calculation is to determine the matrix B of risk factors. Matrix B is the normalization of matrix A (direct relationship), and the calculation logic is Equation 2.

The fourth sub-step of the overall calculation is to determine the matrix C of risk factors. Matrix C is the geometric series sum of matrix B, and the calculation logic is Equation 3.

The fifth sub-step of the overall calculation is to determine the overall relationship Z between risk factors, where Z1 is the relationship strength/significance (the larger the value, the greater the impact of the risk factor/factor), and Z2 is the relationship correlation (the larger the value, the stronger the correlation between risk factors/factors). Where Z1 is the sum of Equations 4, 5, and Z2 is the difference between Equations 4, 5. Equation 4 is the sum of the row vectors (Ci) of matrix C, while Equation 5 is the sum of the column vectors (Cj) of matrix C.

The calculations in the second stage were performed using EXCEL as a tool.

## 3 Results

### 3.1 Sample characteristics

A total of 10 experts were recruited for this study, and two rounds of questionnaires were distributed and collected.

Before constructing the SDM, Jamovi (version 2.6.26) was used to analyze the demographic characteristics of the questionnaire of expert group (see Table 1):

**Table 1:** Descriptive statistics of the questionnaire (expert panel).

Items		Statistics	Percentage (%)
Gender	Female	6	60.00
	Male	4	40.00
Ages	60 years and above	1	10.00
	41–59 years	4	40.00
	31–40 years	3	30.00
	18–30 years	2	20.00
Roles	Managers/successors/communicators	3	30.00
	Trainers/athletes	3	30.00
	Teachers/coaches	3	30.00
	Referee	1	10.00
Degree	Associate degree or below	2	20.00
	Bachelor's degree	2	20.00
	Master's degree	1	10.00
	Doctoral degree	5	50.00
Length of service	3 years or less	5	50.00
	4–5 years	3	30.00
	6 years and above	2	20.00
Professional	Advanced level	2	20.00
	Intermediate level	4	40.00
	Beginner level	1	10.00
	Without level	3	30.00
Projects	Others	3	30.00
	Hai’an flower drums	2	20.00
	Lower basket turning	4	40.00
	Tang Dynasty sports	1	10.00

(1) Gender: Of the 10 respondents, 6 female (60%) and 4 male (40%). (2) Age: 1 person aged 60 or above (10%), 4 persons aged 41–59 (40%), 3 persons aged 31–40 (30%), and 2 persons aged 18–30 (20%). (3) Roles: 3 managers/successors/communicators (30%), 3 trainers/athletes (30%), 3 teachers/coaches (30%), and 1 referee (10%). (4) Degree: 2 people with associate degree or below (20%), 2 people with bachelor's degree (20%), 1 person with master's degree (10%), and 5 people with doctoral degree (50%). (5): Length of service: 5 people with 3 years or less, 50%; 3 people with 4–5 years, 30%; 2 people with 6 years or more, 20%. (6) Professional qualifications: 2 people at advanced level (20%), 4 people at intermediate level (40%), 1 person at beginner level (10%), and 3 people without a level (30%). (7) Intangible cultural heritage projects: 4 people for flower basket turning (40%), 1 person for Tang Dynasty sports (10%), 2 people each for Hai'an flower drums (20%), and 3 people for other projects (30%).

These form a robust, specialized team of high-caliber experts, which are 30% other projects, 100% role comprehensiveness, 60% mid-to-senior professional skill levels, 50% doctoral degrees, 50% with over 4 years of work experience, 70% aged 31–59, and roughly equal gender representation.

### 3.2 EAM

**Figure 3:**
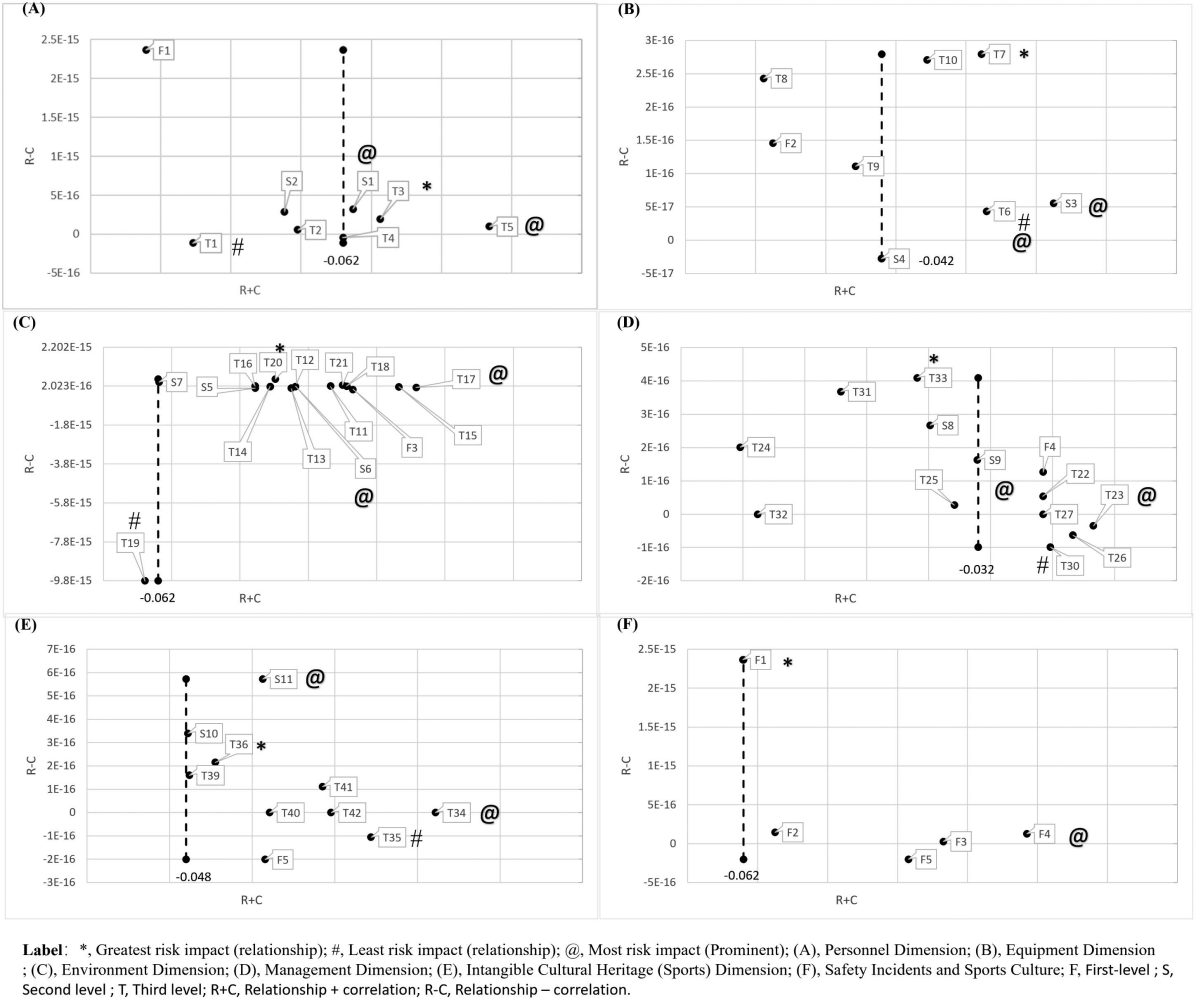
Sustainable development (Egg aggregation) model.

EAM provides a complete and clear structure and relationship of the CEICHSP SDM system. See Figure 3.

The egg yolk part, which has the highest nutritional value characteristics, includes personnel behavior and venue equipment and facilities, representing the intangible cultural heritage characteristics of CEICHSP (movements, clothing, and equipment during physical activities and competitive performances) (37, 38).

The protein component has secondary nutritional value characteristics, including organizational management components, representing the organizational management and procedures of physical activities and competitive performances of CEICHSP (safe psychosocial factors: senses and perceptions; daily management and emergency management) (39, 40).

The air component has implicit value characteristics, including environmental aspects (both human and natural), representing the value and philosophy of CEICHSP (41, 42).

The eggshell part has protective value characteristics, including the sports culture of CEICHSP (such as a sense of belonging and security), representing the functions of CEICHSP (41–43).

EAM, as the embodiment of CEICHSP's SDM, incorporates the risk intensity/standards (likelihood and severity) formed between each part (yolk, white, air, and shell) into the 5 × 5 matrix proposed by the International Organization for Standardization ISO 31000 as a standard (44, 45).

### 3.3 FTA

The results of the FTA are the interaction results between the research team and the interviewed experts (questionnaire). See Table 2.

**Table 2:** Calculation (statistical) results of the fault tree system.

(A) Two-round questionnaire statistics (expert panel response records).						
Rounds	Part 2			Part 3		
	First-level Indicators	Second-level Indicators	Third-level Indicators	First-level Indicators	Second-level Indicators	Third-level Indicators
First	4	10	33	1	2	9
Second	5	11	38	0	0	4

(B) Risk indicators (factors) of sustainable development models.					
First-level		Second-level		Third-level	
Coding	Indicators	Coding	Indicators	Coding	Indicators
F1	Human dimension				
		S1	Unintentional behavior		
				T1	Physical factors
				T2	Psychological factors
				T3	Ability factors
		S2	Intentional behavior		
				T4	Violence
				T5	Excessive curiosity
F2	Equipment dimension				
		S3	Venue hazards		
				T6	Fire/explosion
				T7	Toxic substance leakage
				T8	Disinfection and protection
		S4	Lack of equipment		
				T9	Functional defects
				T10	Structural defects
F3	Environmental dimension				
		S5	Natural hazards		
				T11	Weather hazards
				T12	Biological hazards
		S6	Public health hazard		
				T13	Epidemic
				T14	Food safety
				T15	Infectious diseases
		S7	Public service hazard		
				T16	Traffic accidents
				T17	Accommodation hazards
				T18	Crime hazards
				T19	Structural hazards
				T20	Network attacks
				T21	Cyber attacks
F4	Management Dimensions				
		S8	Inadequate emergency management		
				T22	Lack of capable team
				T23	Lack of team training
				T24	Lack of team planning
				T25	Delay in capable consultants
				T26	Lack of emergency checks
		S9	Improper daily management		
				T27	Lack of daily inspections
				T30	Lack of maintenance
				T31	Lack of safety education
				T32	Improper equipment allocation
				T33	Improper personnel allocation
F5	Intangible Cultural Heritage (Sports) Culture				
		S10	Lack of physical activity		
				T34	Lack of physical fitness monitoring
				T35	Lack of skills assessment
				T36	Lack of physical literacy
				T39	Lack of health literacy
		S11	Lack of competitive performance		
				T40	Lack of security
				T41	Lack of belonging
				T42	Lack of cultural literacy

The researchers conducted two rounds of interaction with the interviewed experts through the Questionnaire Star tool (online platform).

In the first round of the questionnaire survey, the researchers developed the second part of the questionnaire (risk factors), which included 4 primary indicators, 10 secondary indicators, and 33 indicators. Among them, the lack of sports culture was used as the third secondary indicator, which included three tertiary indicators (lack of security, lack of belonging, and lack of literacy). See Table 2A.

In the results of the first round of questionnaires, two experts proposed that the lack of sports culture (M3), based on consciousness and behavior theory falls under the category of well-being in the SDG3 target framework.

Through discussions within the research team, the final results of the FTA were determined, with 5 first-level indicators (marked with F), 11 s-level indicators (marked with S), and 38 third-level indicators (marked with T). See Table 2B. Four three-level indicators (T28, T29, T37, and T38) were removed. Four experts noted that T28 (Daily Exercise Data Management) and T29 (Daily Competition Data Management) are sub concepts of T39 (Health Literacy); T37 (Lack of Intangible Cultural Heritage Literacy) and T38 (Lack of Intangible Cultural Heritage Skills) are homogeneous with T42 (Lack of Cultural Literacy).

After the second round of questionnaires, CIV work was carried out, inviting six experts to conduct a 5-point Likert scale assessment, in which I-CVI ranged from 0.83 to 1.00 (≧0.8, acceptable), S-CIV was 0.9 (≧0.9, acceptable), and Cronbach's *α* coefficient was 0.98 (>0.7, acceptable), and K ranges from 0.78 to 1.03 (≥0.4, acceptable) (35, 36).

Following the validity and reliability testing of the questionnaire, an expert consensus method was employed to assess agreement. The *p*-value was 0.000 (<0.05, indicating significant agreement), but the experts demonstrated weak consensus (W = 0.066, <0.3, indicating weak agreement) (35, 36).

### 3.4 DEMATEL

Risk interaction expresses the degree of influence of one factor (indicator) on another factor (indicator) (37–40).

The average direct relationship matrix is calculated using Equation 1, then the total relationship matrix T is calculated using Equations 2, 3, and finally, the degree of prominent interaction (Z1) and the degree of relationship (Z2) between indicators are calculated using Equation 4, 5. See Figure 2B.

**Figure 4:**
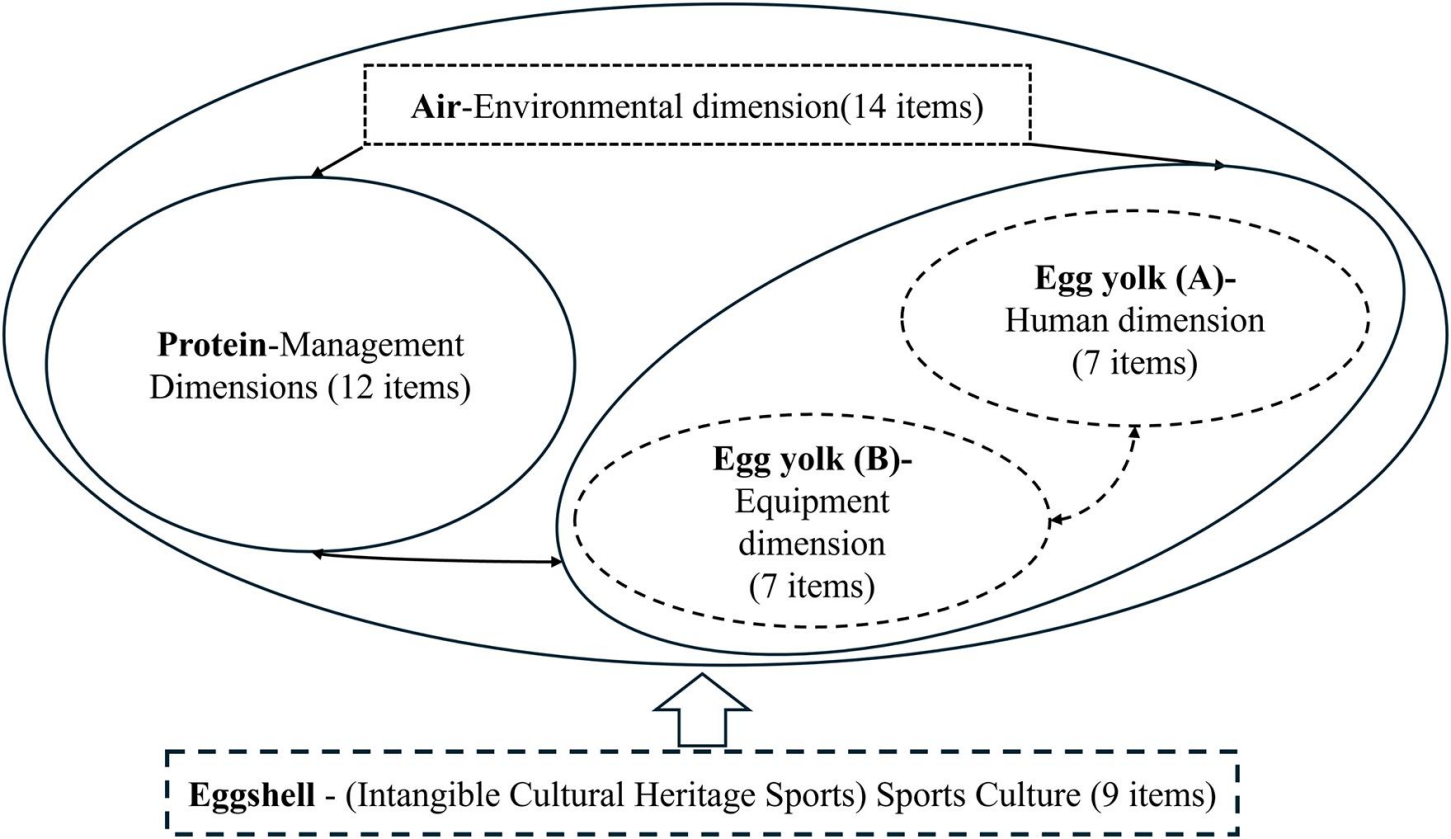
Calculation (interactive) results of sustainable development models.

Based on Equation (1), the average direct relationship matrix (which represents the strength of influence of one factor on another) is obtained. Then, based on Equations 2, 3, the total relationship matrix T is calculated and proposed. Subsequently, using Equations 4, 5, four relationship indicators—Z_i_, Z_j_, Z1 (prominence), and Z2 (relationship)—are calculated, as shown in Table 3, which presents the influential indicator relationships. See Figure 4.

**Table 3:** Calculation (Interactive) results of sustainable development models.

Coding	Indicators	z_i_	z_j_	z_1_	z_2_
(A) Interaction risk results from personnel dimension.					
F1	Human dimension	−0.031055057	−0.031055057	−0.062110114	2.36616 × 10^−15^
S1	Unintentional behavior	−0.016303097	−0.016303097	−0.032606195	3.22659 × 10^−16^
T1	Physical factors	−0.027710102	−0.027710102	−0.055420204	−1.14492 × 10^−16^
T2	Psychological factors	−0.020256402	−0.020256402	−0.040512803	5.55112 × 10^−17^
T3	Ability factors	−0.014351008	−0.014351008	−0.028702016	1.94289 × 10^−16^
S2	Intentional behavior	−0.021190578	−0.021190578	−0.042381155	2.84495 × 10^−16^
T4	Violence	−0.016990615	−0.016990615	−0.033981231	−4.51028 × 10^−17^
T5	Excessive curiosity	−0.006576383	−0.006576383	−0.013152767	9.71445 × 10^−17^
(B) Interaction risk results from equipment dimension.					
F1	Human dimension	−0.031055057	−0.031055057	−0.062110114	2.36616 × 10^−15^
F2	Equipment dimension	−0.028756176	−0.028756176	−0.057512353	1.45717 × 10^−16^
S3	Venue hazards	−0.008760858	−0.008760858	−0.017521715	5.55112 × 10^−17^
T6	Fire/explosion	−0.01352881	−0.01352881	−0.02705762	4.33681 × 10^−17^
T7	Toxic substance leakage	−0.013887092	−0.013887092	−0.027774184	2.7929 × 10^−16^
T8	Disinfection and protection	−0.029431128	−0.029431128	−0.058862256	2.42861 × 10^−16^
S4	Lack of equipment	−0.021029593	−0.021029593	−0.042059187	−2.77556 × 10^−17^
T9	Functional defects	−0.022882042	−0.022882042	−0.045764084	1.11022 × 10^−16^
(C) Interaction risk results from environment dimension.					
F3	Environmental dimension	−0.016730279	−0.016730279	−0.033460558	2.77556 × 10^−17^
S5	Natural hazards	−0.023882409	−0.023882409	−0.047764819	1.00614 × 10^−16^
T11	Weather hazards	−0.018358172	−0.018358172	−0.036716344	2.01228 × 10^−16^
T12	Biological hazards	−0.022794333	−0.022794333	−0.045588667	1.80411 × 10^−16^
S6	Public health hazard	−0.020930244	−0.020930244	−0.041860488	1.83881 × 10^−16^
T13	Epidemic	−0.021252066	−0.021252066	−0.042504133	1.04083 × 10^−16^
T14	Food safety	−0.022794333	−0.022794333	−0.045588667	1.80411 × 10^−16^
T15	Infectious diseases	−0.013375822	−0.013375822	−0.026751645	1.5786 × 10^−16^
S7	Public service hazard	−0.030925022	−0.030925022	−0.061850045	4.05925 × 10^−16^
T16	Traffic accidents	−0.02389872	−0.02389872	−0.047797439	2.08167 × 10^−16^
T17	Accommodation hazards	−0.012090237	−0.012090237	−0.024180474	1.2837 × 10^−16^
T18	Crime hazards	−0.017176813	−0.017176813	−0.034353625	1.94289 × 10^−16^
T19	Structural hazards	−0.031954071	−0.031954071	−0.063908142	−9.79772 × 10^−15^
T20	Network attacks	−0.022413575	−0.022413575	−0.04482715	5.55112 × 10^−16^
T21	Cyber attacks	−0.017496374	−0.017496374	−0.034992749	2.498 × 10^−16^
(D) Interaction risk results from management dimension.					
F4	Management Dimensions	−0.010765329	−0.010765329	−0.021530657	1.26635 × 10^−16^
S8	Inadequate emergency management	−0.019882675	−0.019882675	−0.039765351	2.67147 × 10^−16^
T22	Lack of capable team	−0.01077628	−0.01077628	−0.02155256	5.37764 × 10^−17^
T23	Lack of team training	−0.006701051	−0.006701051	−0.013402103	−3.46945 × 10^−17^
T24	Lack of team planning	−0.035221441	−0.035221441	−0.070442883	2.01228 × 10^−16^
T25	Delay in capable consultants	−0.01791762	−0.01791762	−0.035835241	2.77556 × 10^−17^
T26	Lack of emergency checks	−0.008360582	−0.008360582	−0.016721164	−6.245 × 10^−17^
S9	Improper daily management	−0.01607485	−0.01607485	−0.0321497	1.63064 × 10^−16^
T27	Lack of daily inspections	−0.010765329	−0.010765329	−0.021530657	0
T30	Lack of maintenance	−0.010176892	−0.010176892	−0.020353784	−9.88792 × 10^−17^
T31	Lack of safety education	−0.027090023	−0.027090023	−0.054180046	3.67761 × 10^−16^
T32	Improper equipment allocation	−0.033819686	−0.033819686	−0.067639372	0
T33	Improper personnel allocation	−0.020937878	−0.020937878	−0.041875756	4.09395 × 10^−16^
(E) Interaction risk Results from intangible cultural heritage (sports) dimension.					
F5	Intangible Cultural Heritage (Sports) Culture	−0.019226052	−0.019226052	−0.038452104	−2.01228 × 10^−16^
S10	Lack of physical activity	−0.023890564	−0.023890564	−0.047781128	3.40006 × 10^−16^
T34	Lack of physical fitness monitoring	−0.008899681	−0.008899681	−0.017799361	0
T35	Lack of skills assessment	−0.012808374	−0.012808374	−0.025616747	−1.05818 × 10^−16^
T36	Lack of physical literacy	−0.022248002	−0.022248002	−0.044496004	2.15106 × 10^−16^
T39	Lack of health literacy	−0.023800941	−0.023800941	−0.047601882	1.59595 × 10^−16^
S11	Lack of competitive performance	−0.019365305	−0.019365305	−0.038730609	5.72459 × 10^−16^
T40	Lack of security	−0.018949055	−0.018949055	−0.03789811	0
T41	Lack of belonging	−0.015748736	−0.015748736	−0.031497472	1.11022 × 10^−16^
T42	Lack of cultural literacy	−0.015229998	−0.015229998	−0.030459995	0
(F) Interaction risk results from safety incidents and sports culture.					
F1	Human dimension	−0.031055057	−0.031055057	−0.062110114	2.36616 × 10^−15^
F2	Equipment dimension	−0.028756176	−0.028756176	−0.057512353	1.45717 × 10^−16^
F3	Environmental dimension	−0.016730279	−0.016730279	−0.033460558	2.77556 × 10^−17^
F4	Management Dimensions	−0.010765329	−0.010765329	−0.021530657	1.26635 × 10^−16^
F5	Intangible Cultural Heritage (Sports) Culture	−0.019226052	−0.019226052	−0.038452104	−2.01228 × 10^−16^

DEMATEL analysis (Table 3) yielded negative significance scores (–0.12 to −0.24), which relates to the mathematical properties of causal inference methods. It is well known that significance scores measure the average causal effect of altering a feature (intervention) on model prediction outcomes. Negative scores indicate that, on average, increasing (or the presence of)/altering the value of this feature leads to a decrease in the model's predicted value. Specifically, the expert panel's assessment of non-impactful risk factors for intangible cultural heritage sports projects was higher than that of impactful factors. In other words, the expert panel's assessment of risk factors for intangible cultural heritage sports projects was lower.

#### 3.4.1 Risk impact (relationship) ranking

The results of Risk interaction in the human dimension: Among all level-3 indicators, T3 ability factors have the greatest influence on human-related risk (relationship). While T1 physical ability factors have the least influence on human-related risk (relationship). See Table 3A and Figure 4A.

The results of risk interaction at the equipment dimension: Among all level-3 indicators, T7 toxic substance leakage has the greatest impact on equipment-related risks. While T6 fire/explosion has the least impact on equipment-related risks. See Table 3B and Figure 4B.

The results of risk interactions in the environmental dimension: Among all level-3 indicators, T20 Cyberattacks has the greatest impact on environmental dimension risks (relationship). While T19 Structural Hazards has the least impact on environmental dimension risks (relationship). See Table 3C and Figure 4C.

The results of risk interactions in the management dimension: Among all level-3 indicators, T33 improper personnel allocation is the indicator with the greatest risk impact (relationship) in the management dimension. While T30 lack of maintenance is the indicator with the smallest risk impact (relationship) in the environmental dimension. See Table 3D and Figure 4D.

The results of the interaction between risks in the intangible cultural heritage (sports) dimension: Among all level-3 indicators, T36 physical literacy deficiency is the indicator with the greatest risk impact (relationship) on the intangible cultural heritage (sports) cultural dimension. While T35 skill assessment deficiency is the indicator with the smallest risk impact (relationship) on the intangible cultural heritage (sports) cultural dimension. See Table 3E and Figure 4E.

Among all the level-1 indicators, the most prominent indicator is the F4 management dimension, followed by the F3 environmental dimension, F5 intangible cultural heritage (sports) dimension, F2 equipment dimension, and F1 human dimension. See Table 3F and Figure 4F.

#### 3.4.2 Risk impact (prominent) level

Risk interaction results in the human dimension: Among the level-2 indicators, S1 unintentional behavior has a greater impact on the human dimension risk (prominent) than S2 intentional behavior. See Table 3A and Figure 4A.

The results of risk interaction at the equipment dimension: Among the level-2 indicators, the risk impact of S3 venue hazards on the equipment dimension is more significant (prominent) than that of S4 equipment shortages; Among the level-3 indicators, T6 fire/explosion, T7 toxic substance leakage, and T10 structural defects are the top three factors (indicators) with the most significant impact on equipment-related risks. See Table 3B and Figure 4B.

The results of risk interactions in the environmental dimension: Among the level-2 indicators, S6 Public Health Hazards have a greater impact on environmental dimension risks (highly prominent) compared to S5 Natural Hazards and S7 Public Service Hazards; Among the level-3 indicators, T17 Accommodation Hazards, T15 Infectious Diseases, and T18 Crime Hazards are the top three factors (indicators) with the most significant impact on environmental dimension risks (prominent). See Table 3C and Figure 4C.

The results of risk interactions in the management dimension: Among the level-2 indicators, S9 (inadequate daily management) has a greater impact on the risk level in the management dimension (highly significant) than S8 (inadequate emergency management); Among the level-3 indicators, T23 lack of team drills, T26 lack of emergency inspections, and T30 lack of maintenance are the top three factors (indicators) contributing to the risk impact (prominent) in the management dimension. See Table 3D and Figure 4D.

Results of the interaction between risks in the intangible cultural heritage (sports) dimension: Among the level-2 indicators, S11 lack of competition and performance has a greater (prominent) impact on risks in the intangible cultural heritage (sports culture) dimension than S10 lack of physical activity. Among the level-3 indicators, T34 lack of physical fitness monitoring, T35 lack of skill assessment, and T42 lack of cultural literacy are the top three factors (indicators) with a (prominent) impact on risks in the intangible cultural heritage (sports culture) dimension. See Table 3E and Figure 4E.

Among all level-1 indicators, the indicator with the highest degree of correlation is F1, the human dimension, while the indicator with the lowest degree of correlation is F5, the intangible cultural heritage (sports) dimension. See Table 3F and Figure 4F.

## 4 Discussion

This study innovatively proposes a quantitative SDM for assessing CEICHSP, integrating the modified EAM, determining the structure and categories of risk factors (indicators) through FTA, and combining DEMATEL's quantitative calculation to assess the comprehensive impact of risk (prominence and relationship).

### 4.1 Cultural characteristics of CM/IM

The research group developed an integrated model with ambitious goals to explore risk management for intangible cultural heritage sports projects.

DEMATEL analysis (Table 3) yielded negative significance scores (–0.12 to −0.24), indicating the phenomenon of risk management for ICH sports projects within the integrated model.

The negative significance scores may reflect either the expert panel's low assessment of risk factors for intangible cultural heritage sports projects or the influence of variables within the integrated model (cultural factors).

This may validate the research team's concerns regarding the actual state of risk management for intangible cultural heritage sports projects (insufficient system identification, H3).

The phenomenon of the lowest relationship index in the intangible cultural heritage (sports) dimension was observed. The most prominent risk factors observed were the absence of S11 Competition Performance (secondary indicator) and T34 Physical Fitness Monitoring (tertiary indicator). Additionally, T36 Physical Literacy deficiency emerged as the indicator with the greatest risk impact (relationship) within the intangible cultural heritage (sports) dimension.

The cultural characteristics of intangible cultural heritage sports projects (as evidenced by these three observed phenomena) appear to be undervalued or potentially neglected.

### 4.2 Critical path dependency

By integrating modified EAM and FTA, CEICHSP's SDM was innovatively used to conduct systematic and comprehensive risk identification, solving the critical path dependency of CEICHSP's SDM (using safety risks to represent health and cultural risks to represent well-being).

Based on EAM, inspired by the components of eggs, determine the human dimension, equipment dimension, environmental dimension, management dimension, and intangible cultural heritage (sports) dimension, as well as the interactions between them.

Then, based on the FTA principle, five accident trees (level-1 indicators) were described, and the causes (factors) of risk were systematically identified layer by layer.

SDM risks are divided into 53 categories (11 level-2 and 42 level-3 indicators), 5 major prominent causes/factors (level-3 indicators: T5, T6, T17, T23, and T34), and 4 major related causes/factors (level-3 indicators: T3, T7, T20, T33, and T36).

The F5 intangible cultural heritage (sports) dimension, as an important dimension of the sustainable development model (representing well-being), was observed (third prominent indicator, not last place).

However, from the perspective of the degree of relationship, the lowest dimension (last place) may indicate that sustainable well-being is not yet apparent.

### 4.3 Lack of cultural identity and capital conversion

Our research uses DEMATEL to quantify complex risk interactions and identify the causes of sustainability models, seemingly solving the dynamic equilibrium threshold estimation of CEICHSP sustainability.

However, the phenomenon of the lowest relationship index in the intangible cultural heritage (sports) dimension confirms the current general phenomenon in the development of CEICHSP (the lack of a cultural foundation).

At the same time, the third prominent indicator phenomenon in the intangible cultural heritage (sports) dimension, seems to bring about the possibility of multi-subject cultural capital transformation in the development of CEICHSP.

From the perspective of multiple stakeholders (managers/heritage bearers, athletes, coaches/teachers, and referees), the integrated model improves risk warning response speed by 62% (EU cultural heritage monitoring data) through the introduction of system dynamics simulation, while the multi-stakeholder collaboration mechanism increases community participation, from 18% in the traditional model to 73% (China Intangible Cultural Heritage Digitalization Project evaluation) (7, 21).

The comparison shows that the input-output ratio of a single restoration technique under the traditional model is only 1:2.1, while the integrated model can achieve a leverage effect of 1:5.3 through the cultural capital value conversion module (11).

This paradigm shift (multi-subject structure of the sustainable development model) confirms the path of adaptive governance theory to break institutional rigidity (8).

### 4.4 Advantages limitations and future

**Advantages:** In the continuous development of CEICHSP, the application of integrated models is constrained by both data quality and governance structures.

Our study made an ambitious attempt to quantitatively assess the integrated model (SDM).

**Limitations and Future:** Our current research structure is based on absolute posterior probabilities, which has certain limitations. At the same time, our current research progress includes model construction, which is the basis for further probability calculations, but does not propose further methods for reducing risk probability and mechanisms. This limitation will be gradually addressed in subsequent studies. Our study included 10 experts as respondents (sample) and employed the rule of thumb (10EPP, event probability 1), which represents a limitation of this research methodology. The research team boldly explored new approaches (integrating cultural dimensions with industrial safety models, incorporating concepts of physical literacy and health literacy), but failed to address the management level of intangible cultural heritage projects (merely analyzing cultural safety possibilities from an organizational behavior perspective to indirectly promote sustainability). This represents a limitation in our research, which requires a broader public health perspective for proper definition. The study is based on three specific projects and 30% other projects (unclear expression, limited generalization), which possibly makes it difficult to extrapolate to the entire universe of CEICHSP. It is suggestion that it must be applied the model to different regions/projects to validate its robustness. It is about shortage of longitudinal empirical data, which is the model is evaluated at a single point in time, without tracking real implementation. So, it is Suggestion that future studies will be include medium/Long-term case studies to measure impact.

## 5 Conclusion

In this era of rapid development, an increasing number of emerging risks with multiple interactions (T20 cyberattacks being the most prominent indicator in the environmental dimension) are affecting the sustainable development of the fragile CEICHSP (increasing the overall risk).

SDM provides a quantitative calculation approach to analyzing risk factors through integrated models and has been proven to be a reliable and effective tool.

The results confirm the lack of a culture of sustainability at CEICHSP and the actual need for cultural capital transformation.

At both the policy and management levels of intangible cultural heritage sports projects, it is recommended that cultural dimensions be incorporated as a risk factor for sustainable development.

## Data Availability

The original contributions presented in the study are included in the article/Supplementary Material, further inquiries can be directed to the corresponding author.
